# Laryngeal function-preserving of frontolateral vertical partial laryngectomy (FLVPL) for selected T4a glottic cancer with thyroid cartilage invasion adherence to the anterior commissure: an innovative attempt

**DOI:** 10.1007/s00405-022-07459-8

**Published:** 2022-06-09

**Authors:** Honghong Yan, Di Wu, Jun-hao Mai, Zheng Zhao, Pengfei Xu, Lieqiang Liao, Hongsheng Lin, Xin-rui Zhang, Xue-Kui Liu

**Affiliations:** 1 State Key Laboratory of Oncology in South China, Collaborative Innovation Center for Cancer Medicine, Sun Yat-Sen University Cancer Center, Guangzhou, 510060 Guangdong China; 2 Department of Breast Surgery, Panyu Central Hospital, Guangzhou, China; 3 Department of Otolaryngology Head and Neck Surgery, The Fifth Affiliated Hospital of Guangzhou Medical University, Guangzhou, Guangdong China; 4 Department of Otolaryngology Head and Neck Surgery, The First People’s Hospital of Foshan, Foshan, China

**Keywords:** T4a glottic cancer, Laryngeal framework reconstruction, Titanium mesh

## Abstract

**Objective:**

To evaluate the feasibility and efficacy in selected T4a glottic cancer (thyroid cartilage invasion adherence to the anterior commissure) treated with frontolateral vertical partial laryngectomy (FLVPL) and laryngeal framework reconstruction using titanium mesh.

**Methods:**

Six patients with the limited T4a glottic cancer with thyroid cartilage destruction adherence to the anterior commissure, underwent FLVPL from 2009 to 2016 in Sun Yat-Sen University Cancer Center. All patients were followed up postoperatively.

**Results:**

All patients comprised radical tumor resection and favorable functional outcomes, and no aspiration and laryngeal stenosis were observed. According to postoperative pathology, four patients should go through postsurgical radiotherapy with a mean dose of 66 Gy. But one of them refused to undergo postoperative radiotherapy, who observed local recurrence in postcricoid area underwent total laryngectomy (TL) and ipsilateral selected neck dissection in post-surgery two year. During follow-up period, all patients were still alive, and five patients without local recurrence and distant metastases.

**Conclusion:**

FLVPL and laryngeal framework reconstruction using titanium mesh is one viable surgical procedure to obtain adequate oncologic and functional outcomes.

## Introduction

According to the current guideline T4a laryngeal carcinoma (LC) cases are a specific subgroup where TL is still considered as the main treatment of choice except for selected patients who decline surgery as defined by the National Comprehensive Cancer Network [9]. As the development of material and surgical techniques, some reports have shown that organ preservation surgery may be feasible in a subset of patients with T4a LC. Partial open or transoral robotic laryngectomy may be indicated in some selected “early” T4 laryngeal cancer [6]. Meanwhile, several surgical and options are available for treatment of T4a LC, with comparable results in terms of locoregional control, overall survival (OS), and laryngectomy-free survival [14, 18].

During our observation, a series of T4a patients have confined tumor in a subregion, and the outer cortex of the thyroid cartilage is involved with the tumor through anterior commissure. FLVPL is a surgical treatment option in early glottis LC involving the anterior commissure, and which is grounded on the anatomic and oncologic principles [1, 7]. As a result of tumor causes, vertical cartilage resection requiring the removal of more than half of the vocal folds with anterior commissure is typically large. Primary closure for laryngeal defects after the great cartilage resections is hard and may lead to undesired outcomes such as stenosis. Large defects require suitable reconstruction techniques based on the surgeon's selection and expertise.

Our previously published study [7] showed that titanium mesh was a good alternative for reconstruction of the laryngeal framework, and it provided adequate structural support to maintain airway patency. After gaining experience with early stage cancers, the indication for FLVPL has gradually been extended to the selection (especially anterior commissure involved) T4a glottis LC. Is it possible to perform FLVPL on such patients without compromising survival? Few studies have addressed this issue. This novel technique was approved by our institute’s institutional review board.

## Patients and method

**Fig. 1:**
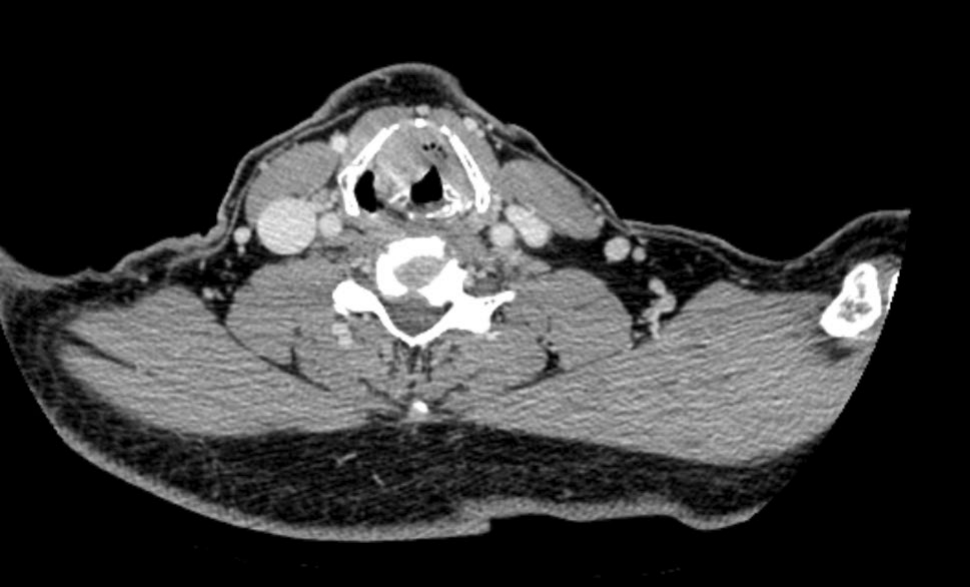
Preoperative enhanced CT images of one of the patients: the laryngeal cancer with slight enhancement has already penetrated and destructed the thyroid cartilage

**Fig. 2:**
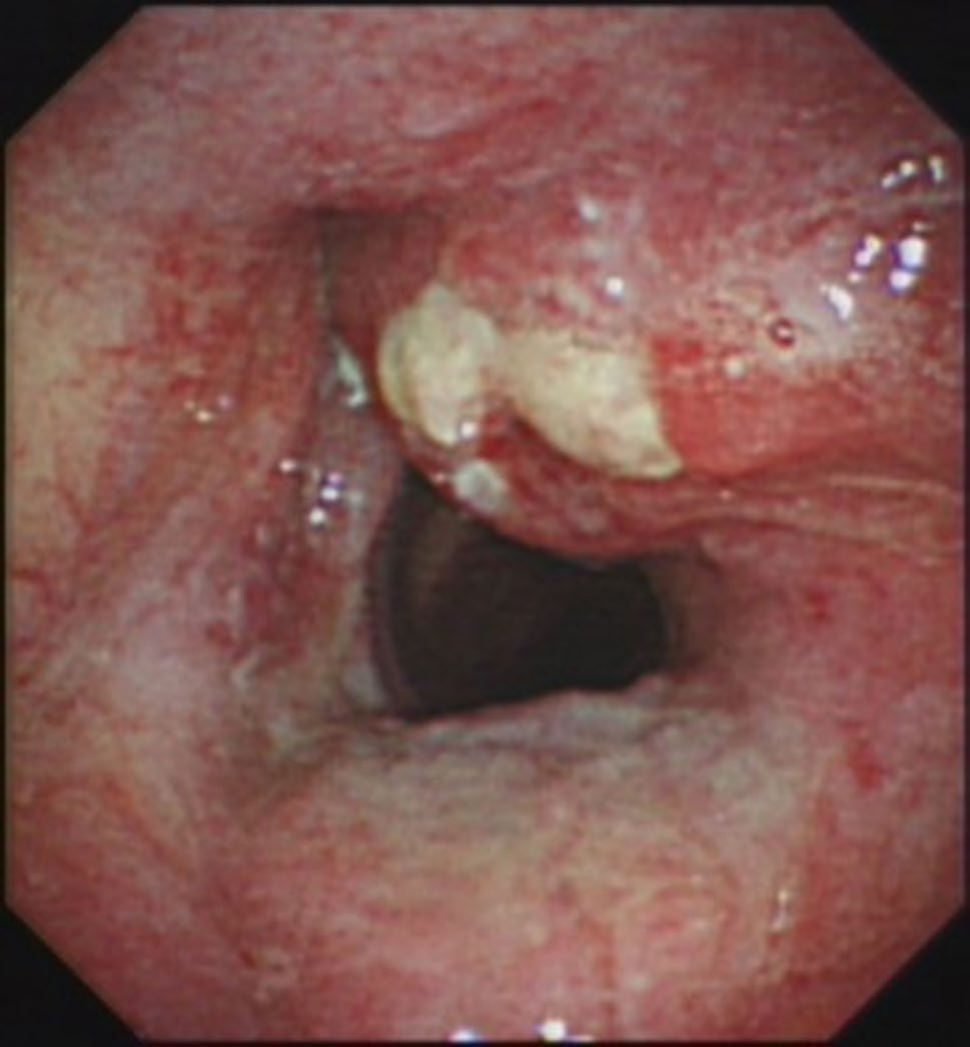
Preoperative endoscopic image of the laryngeal cancer

From 2009 to 2016, we performed extend FLVPL followed by laryngeal framework reconstruction using titanium mesh on 6 patients diagnosed with T4a glottic LC at the Department of Head and Neck Surgery, Sun Yat-Sen University Cancer Center. Depending on the 7th edition of the UICC classification [16], these tumors were classified as T4aN0M0. All of these patients were men, and the mean age was 57 years (range, 42–72 years). All patients were evaluated by thin-section computed tomography (CT) scanning, and endoscopy of the larynx was performed to assess the exact site of the tumor (Figs. 1, 2). The inclusion criteria for this study were: (1) all patients underwent pathological diagnosis of laryngeal squamous carcinoma, and no other treatment was performed before surgery. (2) The tumor is confined to glottic region, with ipsilateral or contralateral vocal cord, anterior commissure, false vocal cord involved, cricoarytenoid joint and epiglottis are not involved. (3) The tumor has caused cartilage invasion and adherence to the anterior commissure (at least outer cortex of the thyroid lamina); normal part of thyroid cartilage is more than one-thirds.

### Operative procedure

The procedure of operation can be divided into two steps: resection and reconstruction. FLVPL is a vertical laryngectomy technique which thyroid cartilage is incised vertically and anterior commissure is resected [7]. After, using a vertical anterior neck incision, separated the strap muscles in the midline, carefully preserve the integrity of muscles. Expose the anterior laryngotracheal wall from hyoid bone to the cricoid cartilage. The isthmus of the thyroid gland was divided.

We enter the larynx from the normal side, passing downward through the false vocal fold, bottom of the ventricle, true vocal fold, and conus elasticus. The resection specimen was retracted to the tumor side, and dissection was continued under visual control across the subglottic region to the lower edge of the arytenoid on the tumor side. The arytenoid was detached and removed, along with the thyroid ala and two vocal folds. On the tumor side, only the epiglottis, the aryepiglottic fold, and about one-quarter of the posterior thyroid cartilage ala were preserved. Resection margins in 3 mm were sent for frozen section evaluation. Additional resection would carry out until the frozen sections showed a negative margin. Neck dissection was depending on the American Academy of Otolaryngology—Head and Neck Surgery Foundation Classification [12]. Adjuvant (chemo)radiotherapy was performed in cases of the pathological examination revealed adverse features or N2 neck disease.

**Fig. 3:**
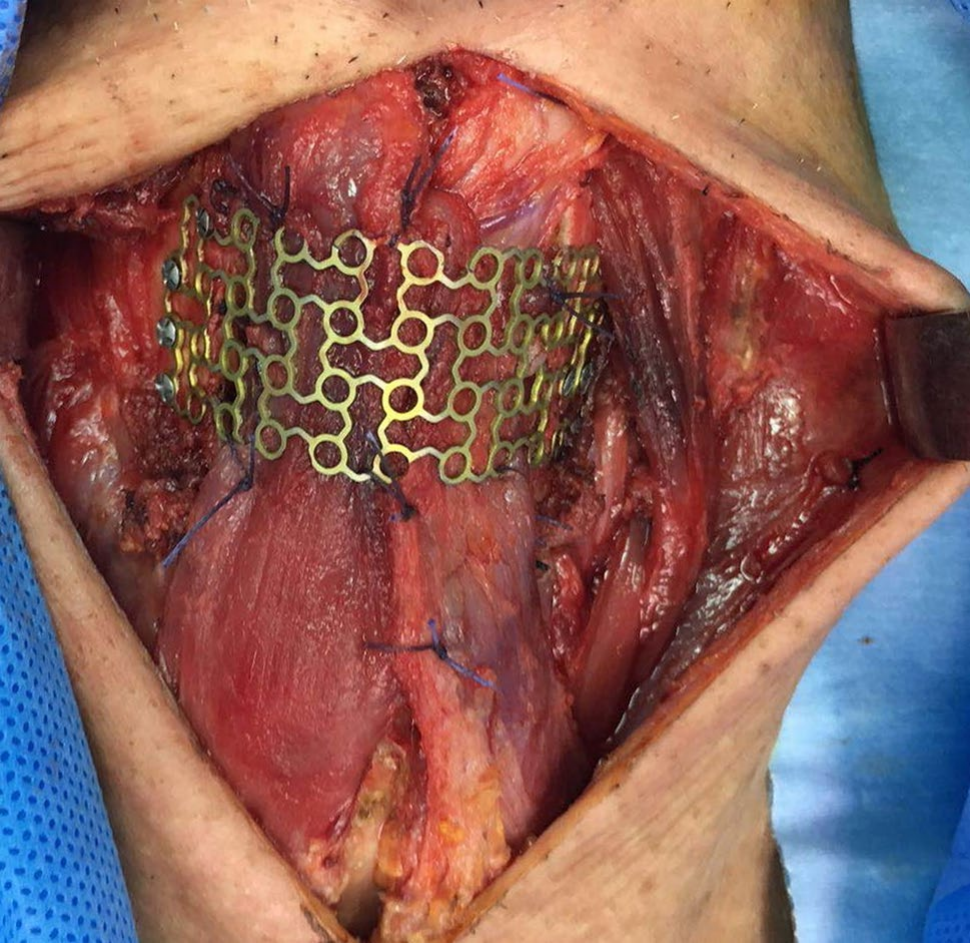
After resection of tumor, the titanium mesh was reshaping, fixed to the remaining thyroid cartilage ala

A suitable titanium mesh (Stryker Leibinger Dynamic Mesh, Freiburg, Germany; 1 mm in thickness) was adapted to the defective area of the thyroid cartilage and then was trimmed to laryngeal framework size with scissors. The trimmed mesh was secured to the reserved thyroid cartilage ala using the appropriate Leibinger bone screws (Fig. 3). The sternohyoid muscles were utilized to cover the inner aspect of the titanium mesh. The omohyoid muscles were used to cover the outer aspect of the mesh. A tracheostomy was done at the end of the procedure. The operative procedure was carried out in all patients.

### Postoperative course

The patient received intravenous injection of antibiotics, corticosteroids for 5 days. Patients had good respiratory function. The trial of decannulation begun gradually by inserting a smaller tracheostomy tube for 24 h, then followed by complete decannulation. On the third postoperative day, the patient was able to consume all food after removing the stomach tube. Swallowing rehabilitation made ordinary meal intake possible. Vocalization to the degree of loud whispering was possible. Pronunciation was clear for daily communication.

Clinical follow-ups were conducted in 2 weeks and every 3 months for the first 3 years post-operation, every 4 and 6 months for the fourth and fifth year, respectively, then continuing with yearly controls. A complete head and neck examination were performed in every clinical visit. Neck ultrasound and a chest X-ray are obtained regularly every 6 months for the initial 3 years.

## Results

### Post-surgical results

The operative procedure was successfully completed in all patients, with no intraoperative or immediate postoperative complications reported. The operative procedure was done with six patients under general anesthesia without tracheostomy, and one patient was performed modified radical neck dissection according to preoperative image findings. All patients got good respiratory function. Decannulation was successful in five patients with tracheostomy five days after operation, and one patient (1/6) was done when postoperative radiotherapy was accomplished. No patients required secondary tracheostomy because of bleeding or laryngeal stenosis. The titanium mesh was well put up with all patients. No titanium mesh was exposed to the larynx lumen.

Oral feeding was found successfully in six patients on the third day postoperatively. Two elderly patients (> 65 years) required temporary nasogastric tube feeding. Two weeks and 3 months postoperatively, CT scanning showed that the titanium mesh was fastened well without displacement and deformity and that there was no laryngeal stenosis. Two weeks postoperatively, fiberscopic inspection showed that the larynx lumen was well maintained. Three months postoperatively, good neo-epithelialization was found on the surface of the strap muscle that used to cover the inner side of the titanium mesh. Only in one patient it was reported that titanium mesh was exposed to the skin after postoperative radiotherapy was finished, which was followed by restoration with adjacent flap.

Postoperatively, all patients have varied degrees of hoarseness. They all can make themselves hear and understood in daily communication. No long-term postoperative complications were recorded.

### Oncologic result

Mean follow-up time was 67.1 months (median, 59; range, 18–160). According to postoperative pathology, four patients should go through postsurgical radiotherapy (including concurrent chemoradiotherapy) with a mean dose of 66 Gy. During the post-radiotherapy period, the titanium mesh was well tolerated and no titanium plate was exposed by all patients. Because the sternohyoid muscles were used to cover the inner aspect of the titanium mesh and the outer aspect of the mesh was covered by omohyoid muscles and skin [7]. But one of them refused to undergo postoperative radiotherapy, who observed local recurrence in postcricoid area underwent TL and ipsilateral selected neck dissection in post-surgery two years. During the follow-up period, all patients were still alive, and five patients without local recurrence and distant metastases.

## Discussion

The use of FLVPL has been internationally accepted as a valid treatment option for early glottic LC involving the anterior commissure and vocal fold mobility, however, its use in advanced LC remains still controversial. In our previous work, we successfully reconstructed defects of laryngeal framework with titanium mesh in patients with T2 or T3 squamous cell carcinoma of glottic after FLVPL. As well as no local recurrence and distant metastasis were found in all patients during follow-up. However, any discussion of oncologic outcomes is challenging and can be descriptive only, since evaluation of the evidence supporting the effectiveness of one treatment over the other is complicated by different outcome measures, use of statistics and investigation of different laryngeal sites [4].

To the best of our knowledge, few data have been published concerning primary surgical therapy in T4a laryngeal carcinoma. Nevertheless, a lack of evidence-based clear-cut consensus still exists on how to differentiate patients with T4a disease amenable to laryngeal conservation surgery from those requiring total laryngectomy [14]. In several studies indicating that organ preservation for larynx cancer was adequate, patients with T4a laryngeal cancer with cartilage invasion [2, 3, 14], even the anterior commissure invasion was included [11]. In other words, the recent evidence for the management of locally advanced glottic laryngeal cancer indicates room for improvement concerning the ideal selection of patients for optimal laryngo-esophageal dysfunction-free and overall survival with tailored multimodal treatment [6].

Anatomically, the anterior commissure of the larynx is generally defined restrictively and functionally as the anterior insertion region of the vocals folds to the thyroid cartilage [11]. Due to the lack of vocal ligament and the inner perichondrium, cartilage’s resistance against tumor invasion is lower and is prone to high incidence of recurrence and so the cartilage adjacent to the tumors involving the anterior commissure must be resected [1]. Recently, the classification of larynx tumors based on compartments has been emphasized as an important point to the indications of partial surgery [10]. From this perspective, the anterior compartments have been shown to be more suitable for the resection of advanced tumors in the case of open partial surgery [5, 14]. Above all, upfront total laryngectomy does not appear to affect the prognosis of T4 laryngeal cancer [8]. This aspect is of outmost relevance in locally advanced tumors where different larynx preservation strategies have been advocated by international guidelines [9]. The delicate tradeoff between oncologic consequences and quality of life requires a more careful and individualized decision-making process in patients with advanced patients. So, selection T4a patients for FLVPL are crucial to obtain adequate oncologic and functional outcomes.

Preservation of the larynx in patients with T4a laryngeal cancer is important. Total laryngectomy is involved in significant morbidity, including creation of a permanent tracheostoma and natural voice loss. Organ preservation is necessarily equivalent to preservation of function and better quality of life. Zhang Caiyun et al. [18] reported 32 patients with T4a laryngeal cancer showed that successful organ preservation surgery(supracricoid subtotal laryngectomy) is safe and reliable, and associated with a relatively acceptable disease-free survival, normal swallowing function and pleasing voice quality. Reconstruction improves postoperative voice function by replacing the soft tissue and relining the lumen of the airway with congratulated epithelium [17]. Like all partial laryngectomies, dysphonia and weakness of the voice are present in FLVPL. Despite having various amounts of deficiencies, all of our patients were able to communicate with an acceptable voice quality in daily life. Furthermore, speech intelligibility after FLVPL seems to be favorable when compared to total laryngectomy even with a voice prosthesis. In brief, low morbidity and mortality and prominent oncologic and functional outcomes validate the importance of FLVPL as an attractive therapeutic option for T4a laryngeal cancer.

Regarding swallowing function, patients who undergo FLVPL for laryngeal cancer usually have a great recovery of deglutition, which compares favorably to open partial laryngectomy [7]. The rate of patients being restricted to soft or liquid food because of swallowing difficulties ranged from 9% to 23% after 1 year, depending on the therapy arm, and 14–16% after 2 years [4]. The median nasogastric tube removal time and decannulation time was 10 and 12 days about the frontal anterior laryngectomy [1]. These figures are much worse than those reported for FLVPL in our present study. Even in our series of locally very advanced laryngeal cancer, the functional outcomes are beneficial. Because of the retention of the epiglottis, oral feeding was recovered successfully on the third day postoperatively. Decannulation was successful in five patients 5 days after operation, and only one patient was done when postoperative radiotherapy was completed.

In carefully selected patients with limited T4a glottic carcinoma with thyroid cartilage destruction adherence to the anterior commissure, FLVPL with or without radiotherapy can be a valuable choice for organ-preserving procedure. In these cases, we reconstructed defects of laryngeal framework with titanium mesh after expansion FLVPL, and titanium mesh could preserve swallowing, speech and respiratory function of the larynx without compromising oncologic results. Depends on the postoperative pathological findings, we also suppose regular postoperative radiotherapy. From an oncological perspective, this technique appears new attempt for selected T4a glottic carcinoma. Although it is the data of only one institution and did not have a large number of patients, we still believe that indications should be strictly controlled before we expand the number, and we will be still able to closely follow up those postoperative patients. Even future well-designed prospective randomized studies are needed.

The NCCN guidelines indicate total laryngectomy as the preferred treatment, particularly for patients with T4a disease [9]. Numerous studies have reported that patients with T4a disease survived better after TL compared with organ-preserving CRT. Timme et al. compared the survival of patients with T3/T4a laryngeal carcinoma patients who underwent primary TL versus those who underwent organ-preserving CRT, and found that the 5-year OS rates were 49% and 16%, respectively [15], other studies reported similar findings [13, 18]. Thus, radical surgery (usually TL) increases the survival of patients with T4a laryngeal cancer. However, in some cases, FLVPL may adequately ensure radical tumor resection. Is it then possible to perform larynx preservation surgery without compromising survival? Few relevant studies have appeared.

## Conclusion

In conclusion, our study suggests that FLVPL and laryngeal framework reconstruction using titanium mesh has an impact on local control and larynx preservation in patients with selected T4a glottic carcinoma. Further prospective studies are necessary to validate our findings and to confirm this surgery procedure might be a recommended option for patients with limited advanced-stage laryngeal cancer.
